# Assessing evidence of inequalities in access to medication for diabetic populations in low- and middle-income countries: a systematic review

**DOI:** 10.3402/gha.v9.32505

**Published:** 2016-12-08

**Authors:** Yodi Christiani, Teerapon Dhippayom, Nathorn Chaiyakunapruk

**Affiliations:** 1 Priority Research Centre for Generational Health and Ageing, Hunter Medical Research Institute, University of Newcastle, Newcastle, Australia; 2 CREDOS (Creative Development Strategies) Institute, Jakarta, Indonesia; 3 Pharmaceutical Care Research Unit, Faculty of Pharmaceutical Sciences, Naresuan University, Phitsanulok, Thailand; 4 Center of Pharmaceutical Outcomes Research, Faculty of Pharmaceutical Sciences, Naresuan University, Phitsanulok, Thailand; 5 School of Pharmacy, Monash University Malaysia, Bandar Sunway, Malaysia; 6 School of Pharmacy, University of Wisconsin, Madison, WI, USA; 7 School of Population Health, University of Queensland, Brisbane, QLD, Australia

**Keywords:** access to medication, diabetes, inequalities, low- and middle-income countries, progress

## Abstract

**Background:**

Inequalities in access to medications among people diagnosed with diabetes inlow- and middle-income countries (LMICs) is a public health concern since untreated diabetes can lead to severe complications and premature death.

**Objective:**

To assess evidence of inequalities in access to medication for diabetes in adult populations of people with diagnosed diabetes in LMICs.

**Design:**

We conducted a systematic review of the literature using the PRISMA-Equity guidelines. A search of five databases – PubMed, Cochrane, CINAHL, PsycINFO, and EMBASE – was conducted from inception to November 2015. Using deductive content analysis, information extracted from the selected articles was analysed according to the PRISMA-Equity guidelines, based on exposure variables (place of residence, race/ethnicity, occupation, gender, religion, education, socio-economic status, social capital, and others).

**Results:**

Fifteen articles (seven quantitative and eight qualitative studies) are included in this review. There were inconsistent findings between studies conducted in different countries and regions although financial and geographic barriers generally contributed to inequalities in access to diabetes medications. The poor, those with relatively low education, and people living in remote areas had less access to diabetes medications. Furthermore, we found that the level of government political commitment through primary health care and in the provision of essential medicines was an important factor in promoting access to medications.

**Conclusions:**

The review indicates that inequalities exist in accessing medication among diabetic populations, although this was not evident in all LMICs. Further research is needed to assess the social determinants of health and medication access for people with diabetes in LMICs.

## Introduction

The 66th World Health Assembly in May 2013 admonished member countries to take action for non-communicable disease (NCD) prevention and control (1). As a result, nine voluntary targets and 25 indicators were highlighted to focus on the key outcomes, risk factors, and national system responses for the prevention and control of NCDs (2). Four of the nine targets are related to diabetes, an indication of the recognition that diabetes is a major NCD. Targets one and seven aim to reduce prevalence and mortality while targets eight and nine aim to improve access to medications, therapy, and counselling for people with diabetes.

In the last few decades, the burden of diabetes has risen globally. An estimated 9% of adults aged 18 years and older had diabetes in 2014, with 80% of these living in low- and middle-income countries (LMICs) (3, 4). Moreover, 1.5 million people died of diabetes-related causes in 2012 with the majority of deaths occurring in LMICs (5). Mortality from diabetes in LMICs is a consequence of high levels of undiagnosed and untreated diabetes (4).

Untreated diabetes can lead to severe complications such as diabetic retinopathy, kidney failure, cardiovascular disease, and premature death. Studies in developed countries have shown that diabetic patients who were older, had high co-morbidity burdens, were from an indigenous group, and had relatively low incomes, were less able to access medications and care (6–8). In LMICs, barriers to diabetes medication include the affordability and availability of essential diabetes medications comprising insulin, glibenclamide, and metformin (9, 10).

The inclusion of diabetes in the global strategy for NCD control and prevention is a signal for governments to strengthen national health systems for the prevention, control, and treatment of diabetes. Where there is an indication of inequality in diabetes care, the coverage of treatment and care should be extended to the population in need. Inequalities in access to medications among people diagnosed with diabetes in LMICs are a public health concern. However, such evidence is lacking. We therefore conducted a systematic review to assess evidence of inequalities in access to medication among adults with diabetes in LMICs. The review is particularly important in identifying population subgroups for targeting interventions so that governments can move towards meeting targets eight and nine of the global strategy.

## Methods

The review was based on the PRISMA-Equity 2012 Statement (Preferred Reporting Items for Systematic Review and Meta-Analysis with a focus on health Equity) (11).

The population of interest was adults aged 18 years or over, who were aware of having been diagnosed with either type 1 diabetes or type 2 diabetes. Cases could have been ascertained either by self-report or clinical measurement. The outcome of interest (untreated diabetes) referred to access to any medications for glycaemic control.

Populations were described by the social determinants of health (place of residence, race/ethnicity, occupation, gender, religion, education, socio-economic status, social capital, and other factors – PROGRESS+) (11). *Place of residence* is an important determinant of health and access to health care. This element of PROGRESS could refer to urban, rural, region, or specific community (e.g. slum), or comparison between countries (12). The *race/ethnicity* component refers to racial, ethnic, cultural background, and language. Although race can be considered as biological determinant, it can determine cultural beliefs and practices that can shape health behaviours including access to health education and care (12). The *occupation* component of PROGRESS comprises different working situations. It includes unemployment, underemployment, informal work, levels of work, and different working environments (12). In this review, gender refers to socially constructed norms and roles in society. *Gender* roles structure various parts of an individual’s life which internalise stereotypical notions of men’s and women’s roles in society that affect their opportunities for health and relationships (13). *Religion* could contribute to inequalities when access to health care is limited because of religious affiliation (12). *Education* is an important determinant of health because of its impact on type of employment and income level (14). *Socio-economic status* is usually determined by income or wealth, which is an important determinant in improving health status through, for example, better living conditions, and access to nutritious food, water, and sanitation and health information (12). In this review, *social capital* is defined as ‘institutions, relationships, and norms that shape the quality and quantity of a society’s social interaction’ (15). Currently there are various approaches to measuring social capital (16–18). The World Bank proposes six main categories for measuring social capital. They are groups and networks, trust and solidarity, collective actions and cooperation, information and communication, social cohesion and inclusion, and empowerment and political action (18). In addition to the PROGRESS components described above, there are *other factors* which may influence health inequalities (referred to as ‘+’ in PROGRESS+). These include age, sexual orientation, disability, and others (19). In this review, we specifically looked at age, disability, and health insurance ownership as the ‘+’ component in assessing inequalities in access to diabetes medications.

### Literature search

We searched the literature from five databases – PubMed, Cochrane, CINAHL, PsycINFO, and EMBASE – from inception to November 2015. The main terms for literature search included ‘Diabetes’, ‘Medication’, ‘Social determinant’, and ‘Low- and middle-income countries’. We adopted the search terms for ‘Medication’, ‘Social determinants’ and ‘Low- and middle-income countries’ from previously published systematic reviews (20, 21). Table 1 gives further details on the terms used in the literature search.

**Table 1:** Search terms^a^

	Search terms
Outcome (access	Diabetes:
to diabetes	diabetes mp. or exp diabetes mellitus/
medication)	diabetes complication mp.
	glycemic index/ or glycemic control/ or glycemic.mp.
	Access to medication^b^:
	pharmaceutical preparations.mp or exp drug/
	pharmaceutical.mp or exp pharmacy/
	medication.mp or exp drug therapy/
	medication.mp or exp medication/
	drug.mp
Exposure	socioeconomic.mp or exp socioeconomics/
(PROGRESS)^c^	inequality.mp or exp social status/ or exp demography/
	inequities.mp or exp health care disparity/
	income.mp or exp lowest income group/ or exp income/ or exp employment status/
	geographic exclusion.mp
	poverty.mp
	residence.mp
	education.mp or exp education/
	ethnic groups.mp or exp racial and ethnic groups/
	migration.mp or exp human migration/
	gender.mp
Population and coverage	developing country.mp or *developing country/
(low- and middle-	developing nation.mp
income	low income countr*.mp
countries)^c^	middle income countr*.mp
	limited resources.mp
	limited setting.mp
	middle east.mp or exp middle east/
	africa.mp or exp africa/
	southeast asia/ or asia.mp. or asia/ or south asia/
	latin america.mp or exp south and central america/
	south america.mp

a Search terms in Ovid. Complete syntax or search terms used in PubMed is attached as the Supplementary file.

b Search terms were developed based on Wirtz et al. (20).

c Search terms were developed based on Langlois et al. (21).

### Inclusion criteria

Studies were included in the review if they 1) examined at least one of the elements in PROGRESS+ as determinant of access to diabetes medication in an adult population (18 years or above), and 2) were conducted in an LMIC category according to the World Bank classification (22). We included both quantitative and qualitative studies in this review and were not restricted by study design. Table 2 gives further detail on the inclusion criteria.

**Table 2:** Inclusion criteria

Inclusion criteria	
Topic	Examining the following exposures: socio-determinant of health, including: place of residence, race/ethnicity, occupation/income level, gender, religion, education, socio-economic status, social groups, marital status, health insurance ownerships, health seeking behaviour, family history of DM (PROGRESS+).
Population	Adults (aged 18 years and older) who have ever been diagnosed with diabetes (type 1 and type 2) by medical professional, either based on their medical record or self-reported; or measured diabetes during survey, but has no access to DM medication (untreated diabetes).
Study outcomes	Receive diabetes medication, or has access to diabetes medication as primary outcome.
Coverage/context	Lower income, lower middle-income, and upper middle-income countries as defined by the World Bank.
Study design	Cross-sectional (single or repeated), cross-country comparison, case-control (prospective or retrospective), cohort studies. We included both quantitative and qualitative approach.

DM, diabetes mellitus.

### Article screening

One reviewer (YC) conducted the literature search. Two independent reviewers (YC and NC) performed the screening and discussed the results. Full texts of articles that passed the screening stage were retrieved, and their eligibility was assessed independently by two reviewers (YC and TD). Any discrepancy in the screening process was resolved through discussion. In the event of lack of consensus on eligibility between two reviewers, the matter was discussed with a third reviewer (NC).

### Quality assessment

Quality assessment for quantitative studies was conducted using the Research Triangle Institute (RTI) Item Bank for Assessing Risk of Bias and Confounding for Observational Studies of Interventions or Exposures (23). Quality assessment for qualitative studies was guided by Cochrane Collaboration Qualitative Methods Group critical appraisal guidelines for qualitative studies (24).

### Data analysis

Information extracted from the included articles was analysed using deductive content analysis while applying the World Health Organization Social Determinants of Health framework (25). We identified PROGRESS+ as predefined categories and developed coding based on these categories. Articles were read, screened, and coded consistent with the predefined codes.

For quantitative studies, data or figures related to these categories were extracted. The extracted information included authors, year of study, study population and sample size, outcome of research, and the findings as they related to PROGRESS+ as the determinants of access to diabetes medications. We extracted 2×2 data or odds ratios for the association of PROGRESS+ and access to medications. The results are presented using a narrative approach according to the PRISMA-E guidelines, based on the exposure (PROGRESS+) (11).

## Results

### Study characteristics

**Fig. 1:**
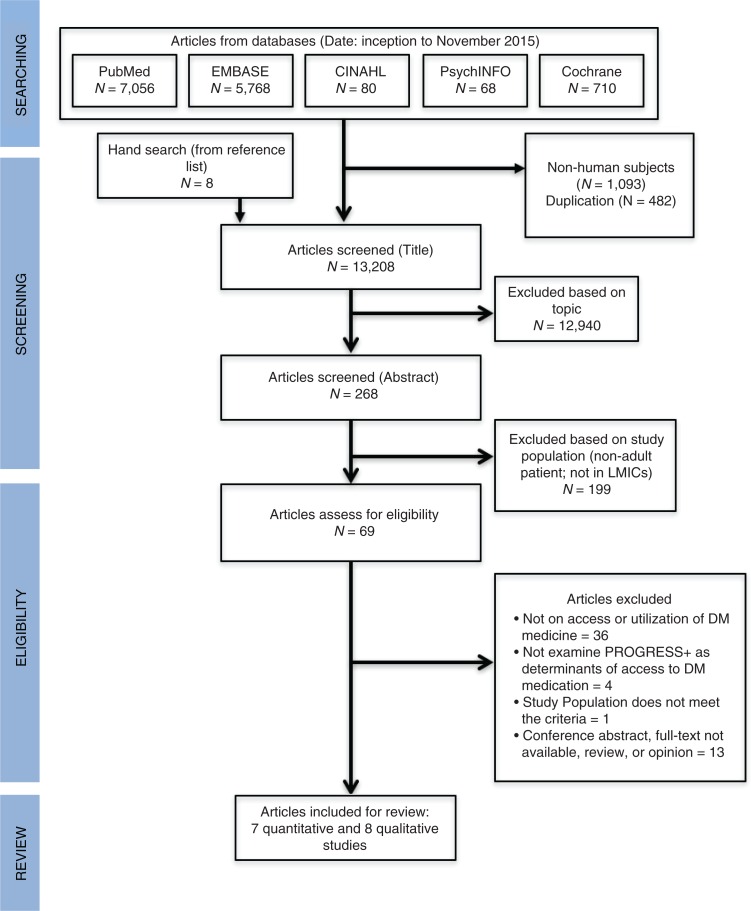
PRISMA flow of studies included.

The literature search from five databases and other sources identified 14,783 articles. In total, 13,208 articles were screened after removing duplicates and studies involving non-human subjects. We excluded 13,139 articles based on screening of titles and abstracts, leaving 69 articles for full text assessment. The review was restricted to manuscripts written in English. In total, 15 articles consisting of eight qualitative (26–33) and seven quantitative studies (34–40) met the inclusion criteria and were included in this review. Figure 1 shows the flow of included studies in this review.

Tables 3 and 4 show summaries of data extracted from the quantitative and qualitative studies, respectively.

**Table 3:** Characteristics of studies included in this review

					Determinants included in the study^a^								
					
Authors	Countries (year^a^)	Study design	Sample size, study population	Outcome	P	R	O	G	R	E	S	S	+
Ben Romdhane et al. (35)	Tunisia (2014)	Quantitative (cross-sectional)	7,700 adults aged 35–70 years old	Prevalence, Awareness, Being untreated	✓		✓	✓		✓	✓		Age
Sosa-Rubi et al. (39)	Mexico (2009)	Quantitative (cross-sectional)	2,960 diabetes patients aged 20–80 years old	Number of insulin injections per week									Health insurance
Stephens et al. (40)	15 LMICs (2013)	Quantitative (cross-sectional)	202,468 prescription	Type of medication prescribed				✓					
Baumann et al. (34)	Uganda (2010)	Quantitative (cross-sectional)	340 diabetes patients aged 30 years or over	Treated, self-management							✓		
Cunningham-Myrie et al. (36)	Jamaica (2013)	Quantitative (cross-sectional)	2,848 adults aged 15 to 74 years old	Prevalence, Awareness, Being treated with any DM medication	✓			✓		✓	✓		Health insurance
Le et al. (38)	Yunan, China (2011)	Quantitative (cross-sectional)	10,007 adults aged 18 years or over in rural Yunan	Prevalence, Awareness, Being treated with any DM medication		✓		✓		✓	✓		Age
Gakidou et al. (37)	Colombia (2007)	Quantitative (cross-sectional)	7,284 adults aged 35–64 years old	Prevalence, Awareness, Being untreated, Being treated and controlled				✓					
	Iran (2004)	Quantitative (cross-sectional)	49,695 adults aged 35–64 years old	Prevalence, Awareness, Being untreated				✓					
	Mexico (1994)	Quantitative (cross-sectional)	30,602 adults aged 35 years or over	Prevalence, Awareness, Being untreated				✓					
	Thailand (2008)	Quantitative (cross-sectional)	33,058 adults aged 35 years or over	Prevalence, Awareness, Being untreated				✓					
Bhojani et al. (28)	India (2013)	Qualitative	16 T2D patients aged 21–65 years old resided in urban slum of Bengaluru	Access to DM medication				✓			✓	✓	
Chary et al. (29)	Guatemala (2012)	Mixed-method	23 indigenous T2D patients resided in indigenous areas of Guatemala aged 18 years or over	Access to DM medication		✓					✓		
Higuchi (30)	The Philippines (2010)	Mixed-method	359 T2D patients, health policy workers, service providers	Access to DM medication, services for DM							✓	✓	Age
Balabanova et al. (26)	Georgia (2008)	Qualitative	14 health policy workers, service providers; and 10 T1D adult patients	Access to insulin	✓						✓		Age
Kolling et al. (31)	Tanzania (2010)	Qualitative	29 T2D patients living in impoverished areas of Dar es Salaam aged 32–70 years old 11 secondary informants (family members, providers, health service manager)	Access to DM medication	✓						✓	✓	Physical condition
Rutebemberwa et al. (33)	Uganda (2013)	Qualitative	32 T2D adults patients (in 4 FGD) in Eastern Uganda and 13 secondary informant	The tendency to use herbal for DM medication	✓						✓	✓	
Belue et al. (27)	Mbour, Senegal (2012)	Qualitative	54 adult diabetic patients attending outpatient clinic	Self-management, being treated							✓	✓	Health insurance

a PROGRESS+: Place of Residence, Race/ethnic, Occupation, Gender, Religion, Education, Socio-economic Status, Social Capital, Others. Check point (✓) indicates determinants included in each study. DM, diabetes mellitus; T1D, type 1 diabetes; T2D, type 2 diabetes; FGD, focus group discussion, LMICs, low- and middle-income countries.

a Year refers to time of data collection.

Studies included in this review were conducted in South America (29, 36, 39), Africa (27, 31, 33–35), Asia (28, 30, 38), Eastern Europe (26, 32), and cross-country studies conducted in different regions (37, 40). The publication years ranged from 2008 to 2014, while data for the secondary analysis by Gakidou et al. (37) were collected between 1994 (in Mexico) and 2008 (in Thailand). We included seven quantitative studies (34–40), six qualitative studies (26–28, 31–33), and two mixed-method studies (29, 30). In the mixed-method studies, only the qualitative components met the inclusion criteria. Thus for the purpose of this review, we refer to these studies (29, 30) as qualitative studies.

The population coverage of the studies was as follows: three quantitative studies (35, 36, 39) covered a national population, two quantitative studies (34, 38), and seven qualitative studies (26–31, 33) covered a sub-national population (specific province or site), two quantitative (37, 40) and one qualitative (32) study covered a cross-countries population. The sample size in the quantitative studies ranged from 2,848 to 49,695 participants (34–39). The qualitative studies included interviews or focus group discussions with 16 to 340 participants (26–33). Additionally, a study by Stephen et al. (40) analysed 202,468 prescriptions for diabetes patients in 15 LMICs.

### Quality assessment

The RTI Item Bank for Assessing Risk of Bias and Confounding for Observational Studies of Interventions or Exposures was used to assess the quality of included studies (23). All quantitative studies in this review applied multistage cluster sampling, which indicated a good representation of the study population. A minimum potential risk of measurement bias was indicated on self-reported access to diabetes medication in six studies (29, 35–39). Table 4 illustrates the quality assessment for quantitative studies included in this review.

**Table 4:** Quality assessment for the quantitative studies included in this review

Studies	Inclusion criteria are varied for each group	Recruitment strategy are varied for each group	Inappropriate comparator group	Valid measures implemented?	Attempt to balance the allocation?	Taking cofounders into account?
Ben Romdhane 2014	N/A	N/A	N/A, study with no comparator group	No, used information from self-reported	N/A	Yes, in the analysis
Sosa-Rubi 2009	No, initially derived data from census	No, original census recruited sample with the same strategy	No, health insurance status is voluntary	No, used information from self-reported	Yes, used standard propensity score matching	Yes, in the analysis
Stephens 2013	N/A	N/A	N/A, study with no comparator group	Yes, IMS prescribing data	N/A	Yes, with age
Cunningham-Myrie 2013	No, initially derived data from health survey	No, original survey recruited sample with the same strategy	No, controls were in accordance with study aim	Cannot determine, reported ‘only current use of pharmacological drugs, was considered as being on therapy’, but didn’t provide detail on how to determine current use	Yes, applied survey weight	Yes, in the analysis
Baumann 2010	N/A	N/A	N/A, study with no comparator group	No, used information from self-reported	N/A	Cannot determine (descriptive results)
Le 2011	N/A	N/A	N/A, study with no comparator group	No, used information from self-reported	N/A	Yes, in the analysis
Gakidoue 2011	N/A	N/A	N/A, study with no comparator group	Cannot determine as measurement approach not reported	N/A	Cannot determine (data were derived from other studies)

N/A, not applicable.

Quality assessment for qualitative studies was guided by the Cochrane Collaboration Qualitative Methods Group critical appraisal guidelines for qualitative studies (24). Study method, approach, design, recruitment strategy, data collection methods, and ethics considerations were appropriate in all the qualitative studies included in this review. Seven out of eight qualitative studies clearly addressed the researcher’s position and included a rigorous data analysis (26–28, 31–33). Further details on quality assessment for qualitative studies are shown in Table 5.

**Table 5:** Quality assessment for the qualitative studies included in this review

Authors	Clearly stated aims	Qualitative method is appropriate	Design appropriate	Recruitment strategy appropriate	Data collection appropriate	Relationships between researcher and participants	Ethical issue	Rigour data analysis	Clear statements of findings	Research is valuable
Bhojani et al. (28)	Yes	Yes	Yes	Yes	Yes	No information	Yes	Yes	Yes	Yes
Chary et al. (29)	Yes	Yes	Yes	No information	Yes	No information	Yes	Yes	Yes	Yes
Higuchi (30)	Yes	Yes	Yes	Yes	Yes	No information	No information	No information	Yes	Yes
Kolling et al. (31)	Yes	Yes	Yes	No information	Yes	Yes	Yes	Yes	Yes	Yes
Balabanova et al. (26)	Yes	Yes	Yes	Yes	Yes	No information	No information	Yes	Yes	Yes
Kühlbrandt et al. (32)	Yes	Yes	Yes	Yes	Yes	Yes	Yes	Yes	Yes	Yes
Rutembemberwa et al. (33)	Yes	Yes	Yes	No information	Yes	No information	Yes	No information	Yes	Yes
Belue et al. (27)	Yes	Yes	Yes	Yes	Yes	No information	Yes	Yes	Yes	Yes

All studies included in this review were cross-sectional studies, which limited our ability to assess the causal relationship between PROGRESS+ and access to diabetes medication.

### Assessing evidence of inequality based on PROGRESS+

None of the studies in this review included all factors in PROGRESS+ in examining access to medication in people with diabetes. The determinants of health evaluated in the included studies were place of residence (*N*=6) (26, 31–33, 35, 36), race/ethnicity (*N*=2) (29, 38), occupation (*N*=1) (35), gender (*N*=7) (28, 31, 35–38, 40), socio-economic status/income (*N*=12) (26–36, 38), and social capital (*N*=6) (27, 28, 30–33). None of the studies examined the association between religion and access to medication. In addition, six studies (26, 30, 31, 35, 38, 39) examined other determinants of access to medication. These included age (*N*=4) (26, 30, 35, 38), physical disability (*N*=1) (31), and health insurance (*N*=3) (27, 36, 39).

#### Place of residence

Two quantitative (35, 36) and four qualitative (26, 31–33) studies examined the association between place of residence and access to diabetes medications (Table 6). Studies conducted in Tunisia (35) and Jamaica (36) revealed no significant association between being untreated and living in an urban area, compared to living in a rural area.

**Table 6:** Main findings of studies included in this review, presented based on the determinants of access to medication among diabetic patients

Determinants	Authors	Country (years)	Study design	Main findings
Place of residence	Ben Romdhane et al.	Tunisia (2014)	Quantitative	The proportion of those who were aware of having diabetes and untreated in urban and rural areas was 11.9 and 11%, respectively (*p*>0.05).
	Cunningham-Myrie et al.	Jamaica (2013)	Quantitative	94.2% of people with diabetes in rural areas were treated, compared to 93.8% in urban areas
	Balabanova et al.	Georgia (2008)	Qualitative	Access to insulin was a problem in rural areas.
	Kolling et al.	Tanzania (2010)	Qualitative	Access to diagnosis and treatment was a problem in rural areas.
	Kühlbrandt et al.	Armenia, Belarus, Moldova, and Ukraine (2014)	Qualitative	Patients in rural areas were disadvantaged in accessing health facilities for screening and treatment by medical professional.
	Rutebenberwa et al.	Uganda (2013)	Qualitative	Patients who had geographical barrier to access health facilities substitute their medication with herbal medication.
Racial/ethnic	Le et al.	Yunan, China (2011)	Quantitative	The minority ethnic group had lower probability to be treated compared to Han (OR=0.26; 95% CI=0.09; 0.73).
	Chary et al.	Guatemala (2012)	Qualitative	In general, indigenous workers received lower payment than other workers. This affected their ability to buy medication for treating DM.
Occupation	Ben Romdhane et al.	Tunisia (2014)	Quantitative	There is no significant association between type of occupation and probability for being untreated.
Gender	Ben Romdhane et al.	Tunisia (2014)	Quantitative	13% of women were untreated compared to 9.6% of men.
	Stephens et al.	15 LMICs (2013)	Quantitative	In Brazil, use of newer drugs were more prevalent for men than women (*p*<0.01).
	Cunningham-Myrie et al.	Jamaica (2013)	Quantitative	There were more women who were treated (95%) compared to men (90.5%).
	Gakidou et al.	Colombia (2007)	Quantitative	16.7% of women and 10% of men who had diabetes were untreated.
		Iran (2004)	Quantitative	11.5% of women and 12.5% of men who had diabetes were untreated.
		Mexico (1994)	Quantitative	2% of women and 4.7% of men who had diabetes were untreated.
		Thailand (2008)	Quantitative	3.2% of women and 8.1% of men who had diabetes were untreated.
	Le et al.	Yunan, China (2011)	Quantitative	17.2% of men and 26.3% of women who had diabetes were treated.
	Bhojani et al.	India (2013)	Qualitative	Domestic roles had restricted women’s access to find medical treatment.
Religion	No studies include religion as determinant of access to diabetes medication			
Education	Ben Romdhane et al.	Tunisia (2014)	Quantitative	There is no significant association between level of education and being untreated.
	Cunningham-Myrie et al.	Jamaica (2013)	Quantitative	There was no significant association between level of education and being treated.
	Le et al.	Yunan, China (2011)	Quantitative	Patients who had primary (OR 2.91; 95% CI=1.69; 4.86) and middle/higher education (OR=2.72; 95% CI=1.22; 4.03) had higher probability to be treated with any DM medication compared to illiterate patients.
Socio-economic status/income	Ben Romdhane et al.	Tunisia (2014)	Quantitative	There are no significant association quintiles of household wealth and being untreated.
	Baumann et al.	Uganda (2010)	Quantitative	37.9% had missed medication because they could not afford it.
	Cunningham-Myrie et al.	Jamaica (2013)	Quantitative	The proportion of people being treated was higher for higher-level income (100%) compared to those with middle-level (92.1%) and lower-level income (91.9%), *p*>0.05.
	Le et al.	Yunan, China (2011)	Quantitative	Those who were categorised as high-income group had higher probability than those in the low-income group (OR=2.92; 95% CI=1.64; 5.57).
	Bhojani et al.	India (2013)	Qualitative	Financial hardships affected people’s access to DM medication. Some of patients reduced their medication dosage or mixed with traditional medication to reduce medication cost.
	Chary et al.	Guatemala (2012)	Qualitative	Among the poor patients, cost of medication is a major barrier for being treated. Some of them bought the prescribed medication only when the household income allowed.
	Higuchi	The Philippines (2010)	Qualitative	Patients expressed financial constraint as major barriers to access or continue DM medication.
	Balabanova et al.	Georgia (2008)	Qualitative	Out-of-pocket payments for insulin acted as a significant barrier to access DM medication.
	Kolling et al.	Tanzania (2010)	Qualitative	Many poor patients were unable to purchase medication.
	Kühlbrandt et al.	Armenia, Belarus, Moldova, and Ukraine (2014)	Qualitative	Out-of-pocket payment for medication was a major barrier for the poor to access medication.
	Rutebenberwa et al.	Uganda (2013)	Qualitative	Patients substituted the medication with herbs because medication was not affordable.
	Belue et al.	Mbour, Senegal (2012)	Qualitative	It is hard for poor patients to get their diabetes treated.
Social capital	Bhojani et al.	India (2013)	Qualitative	Inadequate communication between providers and patients, patients’ negative attitude towards providers, and fragmented nature of health system had limited patient access to medication.
	Higuchi	The Philippines (2010)	Qualitative	Limited local government commitment and budget has affected on low drug availability in public facilities.
	Kolling et al.	Tanzania (2010)	Qualitative	Patients drew supports from their social networks within their local communities to support their medication.
	Kühlbrandt et al.	Armenia, Belarus, Moldova, and Ukraine (2014)	Qualitative	Poorer regions cannot afford to provide free medication. Hence those who resided in those regions had more financial barriers in accessing medication.
	Rutebenberwa et al	Uganda (2013)	Qualitative	Trust to traditional healer increased the tendency of patients to use herbal medication.
	Belue et al.	Mbour, Senegal (2012)	Qualitative	Extended family and the financial systems were associated with diabetes management.
Age	Ben Romdhane et al.	Tunisia (2014)	Quantitative	While it is non-linear, older people with diabetes has lower probability to be untreated compare to those aged 35–39 years old.
	Le et al.	Yunan, China (2011)	Quantitative	Across the age groups, the lowest proportion of people being treated was found in 18–34 years old (5.2%), while the highest prevalence was among those aged 45–54 years old (32.4%).
	Higuchi	The Philippines (2010)	Qualitative	Older patients had less financial support for medication.
	Balabanova et al.	Georgia (2008)	Qualitative	Medication cost is particularly a burden for older people.
Physical condition	Kolling et al.	Tanzania (2010)	Qualitative	Patients with poor physical condition experienced worse financial constrain to afford medication.
Health insurance	Sosa-Rubi et al.	Mexico (2009)	Quantitative	Those who were insured used more insulin per week than those who were not covered by health insurance (13 vs. 9, *p*>0.05).
	Cunningham-Myrie et al.	Jamaica (2013)	Quantitative	100% of people who had health insurance were treated compared to 92.4% of those who had no health insurance.
	Belue et al.	Mbour, Senegal (2012)	Qualitative	Health insurance could benefit access to medication.

DM, diabetes mellitus; LMICs, low- and middle-income countries.

Evidence from qualitative studies revealed contradictory findings. Interviews with health policy workers, health service providers, and managers in Tanzania, Eastern Uganda, and the former Soviet Union countries (26, 31–33) showed people living in rural areas were disadvantaged in accessing diabetes medication. This was particularly the case for those residing in geographically remote areas, due to difficulties in accessing health facilities. People with diabetes in Eastern Uganda chose to substitute biomedication with herbal medication to treat their diabetes (33).

#### Race/ethnicity

One quantitative study (38) and one qualitative study (29) examined the association between ethnicity and access to diabetes medications. In a study conducted in rural southwest China (38), the minority ethnic groups were less likely to be treated with any diabetes medication (OR=0.26; 95% CI=0.09; 0.73). A qualitative study among indigenous groups in Guatemala showed that they had less ability to afford medications to treat diabetes compared with the non-indigenous population, a findings which was related to the lower socio-economic status of the indigenous groups (29).

#### Occupation

One quantitative study assessed the association between employment (upper-level work, middle-level work, and not-working) and access to diabetes medication. This study, conducted in Tanzania, showed no significant association between type of employment and access to diabetes medication (35).

#### Gender

We found five quantitative studies (35–38, 40) and one qualitative study (28) that examined the differences in being treated with any diabetes medication, between men and women.

**Fig. 2:**
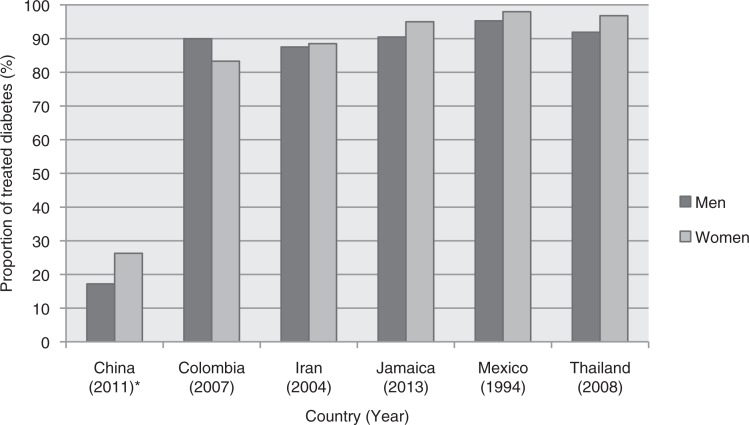
Proportion of treated diabetes, by country for men and women (35–38). *The study in China was a sub-national study conducted in rural areas of Yunan province.

With the exception of Le et al.’s (38) study conducted in rural China, more than 80% of diabetic patients in all other studies had access to medications (35–37). Figure 2 shows the proportion of treated diabetes by country for men and women. In addition, the data show that, women had better access compared to men in most countries except Tunisia and Columbia (35, 37). However, a significant gender inequality was only evident in rural southwest China (38). Despite better access for women in Brazil, men were more likely to be prescribed new or advanced medications for treatment of diabetes compared with women (*p*<0.05) (40).

A qualitative study (28) provided contrasting evidence. Studies conducted among the poor in an impoverished area in Bengaluru, India (28), found that gender roles may limit women’s access to health facilities and diabetes treatment.

#### Religion

We found no study that assessed the association between religion and access to diabetes medications.

#### Education

Two quantitative studies which assessed association between levels of education and access to diabetes medications (35, 38) provided contrasting evidence. In Tunisia, higher levels of education were negatively associated with the probability of being treated with any diabetes medication (although the association was not significant) (35). On the contrary, in rural southwest China those having primary and middle/higher education were more likely to access diabetes medication compared with those who were illiterate (*p*<0.05) (38). We found no qualitative study assessing the association between levels of education and access to medications.

#### Socio-economic status/income

Four quantitative studies (34–36, 38) and eight qualitative studies (26–33) assessed the association between socio-economic status and access to medications.

The two quantitative studies found no significant association between quintiles of household wealth or level of income and access to diabetes medications (35, 36). However, a quantitative study in rural southwest China (38) found high-income groups were more likely to be treated with any diabetes medication compared to low-income groups (OR=2.92; 95% CI=1.64; 5.57). Additionally, 38% of participants in a Ugandan study (34) did not receive medication because of non-affordability.

All qualitative studies reported financial constraints as major barriers in accessing diabetes medication, due to high medical costs (26–33). Even when governments provide free diabetes medications for the poor, in practice, many eligible patients do not have access to these schemes (31). Some people with diabetes who could not afford prescribed medications either partly or fully substituted herbal medications (27, 33). This practice included reducing the prescribed dosage (29), or substituting prescribed medications with more affordable medications (28).

#### Social capital

Although no studies specifically measured social capital, six qualitative studies indicated an association between social capital components and access to diabetes medications (27, 28, 30–33). Firstly, a study in India found that lack of trust in health service providers or the health system had a negative impact on people’s health seeking behaviour, which in turn led to less access to diabetes medications (28).

Secondly, we found three qualitative studies (27, 31, 33), which examined association between social networks and access to medications in diabetic patients. Two qualitative studies conducted in Tanzania (31) and Senegal (27) indicated the importance of support from extended family and friends in diabetes management. Some diabetic patients in impoverished areas drew on support from their friends, colleagues, or people within their local community for their diabetic medication. This applied particularly where there was inadequate support from core or extended family members (31).

A qualitative study in Uganda indicated that social capital could be negatively associated with access to medications among diabetic patients. Living in a neighbourhood with strong beliefs in traditional healing and medication could influence diabetic patients to switch to herbal medications (33).

Thirdly, two qualitative studies (30, 32) indicated an association between political commitment (reflected by government budget allocation) and access to medications. By providing free diabetes medication, the impoverished patients can have access to diabetes medication.

#### Others

In addition to PROGRESS+, six studies (26, 30, 31, 35, 38, 39) examined other determinants of access to diabetes medication. These determinants included age (in four studies) (26, 30, 35, 38), physical disability (in one study) (31), and health insurance (in three studies) (27, 36, 39).

Two quantitative studies (35, 38) and two qualitative studies (26, 30) examined the association between age and access to medications among diabetic patients. The two quantitative studies (35, 38) found that among diabetic patients aged 35 years or over, younger patients had the least access to medications. The proportion of diabetic patients treated was lowest for patients aged 18–34 years (38). In contrast, two qualitative studies found that older people with diabetes had the lowest access to medications due to physical and financial barriers (26, 30).

Two quantitative studies found that people covered by health insurance had better access to diabetes medications than those who were not covered (36, 39). However, this association was not statistically significant (*p*>0.05). A qualitative study in Senegal also showed health insurance provision could improve patient affordability of diabetes medications (27).

We found one qualitative study that showed that diabetic patients with physical disability or in poor physical condition had difficulties in accessing treatment for diabetes (31). These difficulties related to poor physical health and geographical and financial barriers.

## Discussion

Diabetes is one of the main areas of focus in the WHO-Global Strategy for NCD Prevention and Control (1, 2). The burden of diabetes has increased, with the most of the cases and related premature mortality occurring in LMICs. In many LMICs, diabetes has become a public health problem at all levels of socio-economic status (41). Despite high diabetes prevalence and mortality rates in LMICs (5), many people with diabetes are unaware that they have this chronic condition (42).

Access to diabetes medication in LMICs is rarely assessed. In an attempt to address this gap, we conducted a review of published articles to assess inequalities in access to diabetes medication in LMICs. The studies included in this review have a minimum risk of bias and good representation of the study population.

In general, the barriers in accessing diabetes medications in LMICs include affordability, lack of access to health care, poor diagnostic and monitoring equipment, and lack of trained health workers to provide treatment (9). This systematic review indicates the existence of inequalities in access to diabetes medications, although conditions varied across countries. We focused our review on the social determinants of health (place of residence, race/ethnicity, occupation, gender, religion, education, socio-economic status, social capital, and others – PROGRESS+) in relation to the access and utilisation of diabetes medication among adult diabetic patients. Although our findings may not be applicable to all LMICs, there is an indication that diabetic patients who resided in rural or remote areas (31–33), minority ethnic group/indigenous populations (29, 38), women (28, 31, 40), those with low education (38), and of low-income/social-economic status (27–33) were disadvantaged with respect to accessing diabetes medications. The findings indicated the importance of addressing the social determinants of health in improving access to medication and health care.

Governments’ commitments to providing essential medications in primary care are crucial. Furthermore, the relationship between health workers and patients regarding ongoing treatment is also important (26, 30, 32). These are areas which need to be addressed in order to reduce inequalities in access to diabetes medications.

An unexpected finding was that few studies have closely examined access to medication among diabetic patients in LMICs. Most epidemiological studies on diabetes in LMICs have broadly focused on prevalence of diagnosed and undiagnosed diabetes (43–48), and the effect of treatment (i.e. controlled diabetes) (49–55). Target nine of the global strategy refers to providing access to essential medicines for NCDs in 80% of the population. In studies covering national populations in LMICs, more than 80% of people diagnosed with diabetes had access to medications (35–37). However, because of the high rate of undiagnosed diabetes in LMICs (4), it is plausible that a huge proportion of people with diabetes are left undiagnosed and untreated. Hence the 80% target is well short of being achieved.

Additionally, we found inconsistent results across the studies. This variation in results arose from different population coverage (national, sub-national, and certain group), heterogeneity in health care systems and health insurance coverage, and differences in data sources (household survey, interview with policy workers, and prescription for diabetes patient). For example, two nationwide studies found that people residing in urban and rural areas had the same level of access to medication (35, 36). However, studies conducted in particular settings (rural, indigenous areas) found that diabetic patients residing in rural and remote areas had less access to medication (29, 33, 38). The situation is of particular concern given type 2 diabetes has become a major health issue in rural areas in LMICs (4).

Our review provides several implications for future direction. First, inequality in access to medication can stem from both structural factors (place of residence, age, gender, education, and socio-economic status) and intermediary determinants (social capital, health system, and health care provision). In this review, there is an indication that the poor were disadvantaged in accessing medication because of high costs of medications. People with chronic diseases, such as diabetes, cardiovascular disease, and asthma require long-term therapies. However, access to needed medications is still limited in many LMICs (56). In this respect there is a need for a pro-poor policy, providing essential medication at affordable costs. One of the strategies is promoting the use of generic medications and improving medication availability in the public sector (57). This is particularly important in rural and remote areas, where the health workers’ capacity for providing treatment and care for people with diabetes needs to be strengthened. In the broader context, inequality in socio-economic conditions should be addressed.

The review found that national health systems can play critical roles in reducing inequalities of access to medications. In LMICs, inadequate political commitment to NCDs may underestimate the threat of diabetes (58, 59). This in turn could lead to poor diabetes prevention, screening, and treatment services (59). Strong government commitment through sufficient budget allocation in medications and health insurance provision could improve access to health care. Nevertheless, the impact will be suboptimal unless there is strengthening of primary care services (28, 30, 31).This includes developing public trust with health service providers and the health system through both public education and capacity building for health workers in providing good care for their patients.

Finally, there were limited studies on access to medications among diabetic patients. Given the high burden of diabetes, it is important to conduct studies on the determinants of access to medications among diabetic patients, particularly in LMICs. Financial and geographic barriers were two common factors underlying inequalities. Both national and sub-national studies are needed in LMICs, where there are wide social and economic disparities across regions.

### Study limitation

The literature included in this review was restricted to online database and peer-review articles. Our search terms were adopted from previous studies (20, 21). Although it may not cover all words used in PROGRESS+ elements, we believe that, it is specific to the outcome and exposures of interest. The limited number of studies included in this review did not allow us to discuss each essential medication (insulin, glibenclamide, and metformin) separately. Despite these limitations, our findings indicate the existence of socio-economic inequalities in access to diabetes medications in LMICs and identify population subgroups who need to be targeted in order to reduce these inequalities.

## Conclusions

We found few studies examining access to medications among diabetic patients in LMICs. The determinants of access to medication varied across countries and study settings. The results of this review should be interpreted in light of the heterogeneity in study settings, design, sample size, participants, and importantly contextual factors such as the culture and national health system within countries. In summary, this review indicates inequalities in access to medications among people diagnosed with diabetes in LMICs, although this was not evident in all LMICs. However, inequalities extend beyond those identified here as large numbers of people with diabetes in LMICs remain undiagnosed and untreated.

## Supplementary Material

Assessing evidence of inequalities in access to medication for diabetic populations in low- and middle-income countries: a systematic review

Assessing evidence of inequalities in access to medication for diabetic populations in low- and middle-income countries: a systematic review

## Paper context

Diabetes is a major public health problem in LMICs, with many undiagnosed and untreated cases. This review assesses evidence of inequalities in access to diabetes medications among adults known to have diabetes. Barriers of affordability and availability result in unequal access within and across populations. There is a need for stronger government political commitment to improve access to diagnosis and treatment for diabetes in LMICs.
