# Supplementary material for: Assessing evidence of inequalities in access to medication for diabetic populations in low- and middle-income countries: a systematic review
Source: Glob Health Action. 2016 Dec 8;9:10.3402/gha.v9.32505. doi: 10.3402/gha.v9.32505 (PMC5148807; doi:10.3402/gha.v9.32505)
Supplement: Assessing evidence of inequalities in access to medication for diabetic populations in low- and middle-income countries: a systematic review [file GHA-9-32505-s001.pdf]

Keywords

Diabetes
(("diabetes mellitus"[MeSH Terms] OR ("diabetes"[All Fields] AND "mellitus"[All Fields]) OR "diabetes mellitus"[All Fields]) OR ("diabetes complications"[MeSH Terms] OR ("diabetes"[All Fields] AND "complications"[All Fields]) OR "diabetes complications"[All Fields])) OR glycemic[All Fields]

AND

Access to Medicine[1]
((("pharmaceutical preparations"[MeSH Terms] OR ("pharmaceutical"[All Fields] AND "preparations"[All Fields]) OR "pharmaceutical preparations"[All Fields] OR "drugs"[All Fields]) OR ("pharmaceutical preparations"[MeSH Terms] OR ("pharmaceutical"[All Fields] AND "preparations"[All Fields]) OR "pharmaceutical preparations"[All Fields] OR "medication"[All Fields])) OR ("medicine"[MeSH Terms] OR "medicine"[All Fields])) OR ("pharmacy"[MeSH Terms] OR "pharmacy"[All Fields] OR "pharmacies"[MeSH Terms] OR "pharmacies"[All Fields])

Social Determinant[2]
(("socioeconomic factors"[MeSH Terms] OR ("socioeconomic"[All Fields] AND "factors"[All Fields]) OR "socioeconomic factors"[All Fields] OR "inequality"[All Fields]) OR ("socioeconomic factors"[MeSH Terms] OR ("socioeconomic"[All Fields] AND "factors"[All Fields]) OR "socioeconomic factors"[All Fields] OR "inequalities"[All Fields]) OR equity[All Fields] OR inequity[All Fields] OR inequities[All Fields] OR socio-economic[All Fields] OR ("socioeconomic factors"[MeSH Terms] OR ("socioeconomic"[All Fields] AND "factors"[All Fields]) OR "socioeconomic factors"[All Fields] OR "inequalities"[All Fields]) OR (socio-economic[All Fields] AND ("socioeconomic factors"[MeSH Terms] OR ("socioeconomic"[All Fields] AND "factors"[All Fields]) OR "socioeconomic factors"[All Fields] OR "inequality"[All Fields])) OR (socio-economic[All Fields] AND inequities[All Fields]) OR (socio-economic[All Fields] AND inequity[All Fields]) OR (socioeconomic[All Fields] AND ("socioeconomic factors"[MeSH Terms] OR ("socioeconomic"[All Fields] AND "factors"[All Fields]) OR "socioeconomic factors"[All Fields] OR "inequalities"[All Fields])) OR (socioeconomic[All Fields] AND ("socioeconomic factors"[MeSH Terms] OR ("socioeconomic"[All Fields] AND "factors"[All Fields]) OR "socioeconomic factors"[All Fields] OR "inequality"[All Fields])) OR (socioeconomic[All Fields] AND inequities[All Fields]) OR (socioeconomic[All Fields] AND inequity[All Fields]) OR (unmet[All Fields] AND need[All Fields]) OR barrier[All Fields] OR ("income"[MeSH Terms] OR "income"[All Fields]) OR ("socioeconomic factors"[MeSH Terms] OR ("socioeconomic"[All Fields] AND "factors"[All Fields]) OR "socioeconomic factors"[All Fields] OR "socioeconomics"[All Fields]) OR geographic[All Fields] OR exclusion[All Fields] OR ("poverty"[MeSH Terms] OR "poverty"[All Fields]) OR vulnerability[All Fields] OR

---

[1] Keywords are developed based on Wirtz VJ, Reich MR, Leyva-Flores R, Dreser A. "Medicines in Mexico, 1990-2004: systematic review of research on access and use". *Salud Publica Mex* 2008;50 suppl 4:S470-S479.

[2] We follow the keywords used in Langlois, Étienne V., et al. "Inequities in postnatal care in low-and middle-income countries: a systematic review and meta-analysis." *Bulletin of the World Health Organization* 93.4 (2015): 259-270G.

marginalized[All Fields] OR vulnerable[All Fields] OR marginalization[All Fields] OR ("social distance"[MeSH Terms] OR ("social"[All Fields] AND "distance"[All Fields]) OR "social distance"[All Fields] OR ("social"[All Fields] AND "exclusion"[All Fields]) OR "social exclusion"[All Fields]) OR (social[All Fields] AND stratification[All Fields]) OR gradient[All Fields] OR determinant[All Fields] OR predictor[All Fields] OR propension[All Fields] OR ("socioeconomic factors"[MeSH Terms] OR ("socioeconomic"[All Fields] AND "factors"[All Fields]) OR "socioeconomic factors"[All Fields]) OR ("risk factors"[MeSH Terms] OR ("risk"[All Fields] AND "factors"[All Fields]) OR "risk factors"[All Fields] OR ("risk"[All Fields] AND "factor"[All Fields]) OR "risk factor"[All Fields]) OR residence[All Fields] OR location[All Fields] OR ("ethnology"[Subheading] OR "ethnology"[All Fields] OR "ethnicity"[All Fields] OR "ethnology"[MeSH Terms] OR "ethnicity"[All Fields] OR "ethnic groups"[MeSH Terms] OR ("ethnic"[All Fields] AND "groups"[All Fields]) OR "ethnic groups"[All Fields]) OR (("emigration and immigration"[MeSH Terms] OR ("emigration"[All Fields] AND "immigration"[All Fields]) OR "emigration and immigration"[All Fields] OR "immigration"[All Fields]) AND status[All Fields]))

AND

LMICs[3]
(((("developing countries"[MeSH Terms] OR ("developing"[All Fields] AND "countries"[All Fields]) OR "developing countries"[All Fields]) OR ("developing countries"[MeSH Terms] OR ("developing"[All Fields] AND "countries"[All Fields]) OR "developing countries"[All Fields] OR ("developing"[All Fields] AND "country"[All Fields]) OR "developing country"[All Fields]) OR ("developing countries"[MeSH Terms] OR ("developing"[All Fields] AND "countries"[All Fields]) OR "developing countries"[All Fields] OR ("underdeveloped"[All Fields] AND "countries"[All Fields]) OR "underdeveloped countries"[All Fields]) OR ("developing countries"[MeSH Terms] OR ("developing"[All Fields] AND "countries"[All Fields]) OR "developing countries"[All Fields] OR ("underdeveloped"[All Fields] AND "country"[All Fields]) OR "underdeveloped country"[All Fields]) OR (emergent[All Fields] AND countries[All Fields]) OR (emergent[All Fields] AND country[All Fields]) OR ("developing countries"[MeSH Terms] OR ("developing"[All Fields] AND "countries"[All Fields]) OR "developing countries"[All Fields] OR ("developing"[All Fields] AND "nation"[All Fields]) OR "developing nation"[All Fields]) OR (underdeveloped[All Fields] AND "nation"[All Fields])) OR (emergent[All Fields] AND "nation"[All Fields]) OR ("africa"[MeSH Terms] OR "africa"[All Fields]) OR (("poverty"[MeSH Terms] OR "poverty"[All Fields] OR ("low"[All Fields] AND "income"[All Fields]) OR "low income"[All Fields]) AND countries[All Fields]) OR (("poverty"[MeSH Terms] OR "poverty"[All Fields] OR ("low"[All Fields] AND "income"[All Fields]) OR "low income"[All Fields]) AND country[All Fields]) OR (middle[All Fields] AND ("income"[MeSH Terms] OR "income"[All Fields]) AND countries[All Fields]) OR (middle[All Fields] AND ("income"[MeSH Terms] OR "income"[All Fields]) AND country[All Fields]) OR (("poverty"[MeSH Terms] OR "poverty"[All Fields] OR "poor"[All Fields]) AND setting[All Fields]) OR (("health resources"[MeSH Terms] OR ("health"[All Fields] AND "resources"[All Fields]) OR "health resources"[All Fields] OR "resource"[All Fields]) AND limited[All Fields] AND setting[All Fields]) OR (("health resources"[MeSH Terms] OR ("health"[All Fields] AND "resources"[All Fields]) OR "health resources"[All Fields] OR "resource"[All Fields]) AND scarce[All Fields] AND setting[All Fields]) OR (resource-limited[All Fields] AND setting[All Fields]) OR (resource-scarce[All Fields] AND setting[All Fields]) OR (("poverty"[MeSH Terms] OR "poverty"[All Fields] OR "poor"[All Fields]) AND country[All Fields]) OR (("poverty"[MeSH Terms] OR "poverty"[All Fields] OR ("low"[All Fields] AND "income"[All Fields]) OR "low income"[All Fields]) AND "nation"[All Fields]) OR (middle[All

[3] We follow the keywords used in Langlois, Étienne V., et al. "Inequities in postnatal care in low-and middle-income countries: a systematic review and meta-analysis." *Bulletin of the World Health Organization* 93.4 (2015): 259-270G.

Fields] AND ("income"[MeSH Terms] OR "income"[All Fields]) AND "nation"[All Fields]) OR (third[All Fields] AND "world"[All Fields]) OR ("middle east"[MeSH Terms] OR ("middle"[All Fields] AND "east"[All Fields]) OR "middle east"[All Fields]) OR ("india"[MeSH Terms] OR "india"[All Fields]) OR ("asia"[MeSH Terms] OR "asia"[All Fields]) OR ("europe, eastern"[MeSH Terms] OR ("europe"[All Fields] AND "eastern"[All Fields]) OR "eastern europe"[All Fields] OR ("eastern"[All Fields] AND "europe"[All Fields])) OR ("philippines"[MeSH Terms] OR "philippines"[All Fields]) OR ("indonesia"[MeSH Terms] OR "indonesia"[All Fields]) OR ("latin america"[MeSH Terms] OR ("latin"[All Fields] AND "america"[All Fields]) OR "latin america"[All Fields]) OR ("south america"[MeSH Terms] OR ("south"[All Fields] AND "america"[All Fields]) OR "south america"[All Fields]) OR ("central america"[MeSH Terms] OR ("central"[All Fields] AND "america"[All Fields]) OR "central america"[All Fields]) OR ("china"[MeSH Terms] OR "china"[All Fields]) OR ("russia"[MeSH Terms] OR "russia"[All Fields]))
